# Antitumor activity of Koningic acid in thyroid cancer by inhibiting cellular glycolysis

**DOI:** 10.1007/s12020-021-02822-x

**Published:** 2021-07-15

**Authors:** Changxin Jing, Yanyan Li, Zhifei Gao, Rong Wang

**Affiliations:** 1 Department of Endocrinology, The First Hospital of Yulin, 719000 Yulin, PR China; 2 Department of Endocrinology, The First Affiliated Hospital of Xi’an Jiaotong University, 710061 Xi’an, PR China; 3 Key Laboratory for Tumor Precision Medicine of Shaanxi Province, The First Affiliated Hospital of Xi’an Jiaotong University, 710061 Xi’an, PR China

**Keywords:** Koningic acid, Thyroid cancer, Glycolysis ability, Antineoplastic activity, Extracellular acidification rate, ATP deprivation

## Abstract

**Purpose:**

Koningic acid (KA), a sesquiterpene lactone, has been identified as an antimicrobial agent. Recent studies have revealed KA’s antitumor activities in colorectal cancer, leukemia, and lung cancer. However, its antitumor effect in thyroid cancer remains largely unknown.

**Methods:**

The effects of KA on proliferation, colony formation, apoptosis in thyroid cancer cells were assessed by MTT assay and flow cytometry. After KA treatment, the glycolysis ability of thyroid cancer cells was detected by ECAR, and the glycolytic products and relative ATP levels were measured by ELISA. The underlying mechanisms of antineoplastic activity of KA in thyroid cancer were detected by Western blot. Finally, the antineoplastic activity in vivo was observed in Xenograft mouse models.

**Results:**

KA inhibited the proliferation, colony formation, and increased cell apoptosis in thyroid cancer cell lines in a dose and time-dependent manner. We verified that the glycolysis ability, ATP production, and lactic acid level in thyroid cancer cells had experienced an extensive decrease after KA treatment. In addition, lactic acid, the metabolite of glycolysis, could weaken the effect of KA on its colony formation ability in C643 thyroid cancer cell line. Our data also showed that KA kills thyroid cancer cells by inhibiting the MAPK/ERK pathway and decreasing Bcl-2 level. By contrast with the control group, the growth of xenograft tumor was dramatically inhibited by KA without obvious drug side effects.

**Conclusion:**

Our data demonstrate that KA kills thyroid cancer cell lines by inhibiting their glycolysis ability, the MAPK/ERK pathway and the Bcl-2 level and suggest that KA has potential clinical value in thyroid cancer therapy.

## Introduction

Thyroid cancer is a common endocrine malignancy which has a rapidly increased in global incidence in recent decades. The global incidence of thyroid cancer in women is 10.2 per 100,000, which is 3 times higher than in men [1]. Thyroid cancer is classified into differentiated and undifferentiated thyroid cancer. The former consists of papillary thyroid cancer and follicular thyroid cancer, which account for the majority of thyroid cancers [2]. The mortality rates of thyroid cancer are low, with rates from 0.4 to 0.5 in men and women, respectively, and its prognosis is good. However, the rate of recurrence or persistence in thyroid cancer is high and there is risk of developing into anaplastic thyroid cancer (ATC). Although ATC is a rare type of undifferentiated thyroid cancer that makes up ~1% of thyroid cancer cases, it is one of the most lethal malignant tumors in human being [3–5]. Conventional surgical thyroidectomy with adjuvant ablation by radioiodine treatment has been the mainstay of thyroid cancer treatment. However, it is often not curative for patients diagnosed with ATC. ATC is one of the most aggressive and lethal human cancers due to its invasive growth behavior and high propensity for distant metastasis [6]. Therefore, improving therapeutic strategy against thyroid cancer, especially ATC, is urgently in need.

One hallmark of the rapidly proliferating tumor is a shift from mitochondrial respiration to aerobic glycolysis (Warburg effect) [7]. Although aerobic glycolysis is inefficient from an energy production perspective, it can provide the required biomass for the rapid proliferation [8]. The distinct metabolism of tumor cells makes targeting metabolic pathways as a promising approach for therapeutic interventions.

Koningic acid (KA), also known as heptelidic acid, is a sesquiterpene lactone initially identified as an antimicrobial agent, active against anaerobic bacteria and displays antiparasitic properties [9, 10]. Earlier studies have confirmed that KA is an effective and specific inhibitor of glyceraldehyde 3-phosphate dehydrogenase (GAPDH), inactivates GAPDH via covalent binding to a cysteine residue in the active site of the enzyme, which is known to be a glycolytic catalytic enzyme and affects the synthesis of ATP [11–14]. It has been demonstrated that KA selectively kills high-glycolytic cells through glucose-dependent active ATP deprivation [15]. This mechanism may provide an effective treatment for cancer that rely on high glucose metabolism. However, until now, its antineoplastic activity in thyroid cancer remains largely unknown. In this study, we demonstrated that KA could effectively kill thyroid cancer cells depending on its suppression ability of glycolysis, ATP production and the MAPK/ERK signaling pathways, thereby impeding the malignant progression of thyroid cancer.

## Materials and methods

### Thyroid cancer cell lines

Human immortalized thyroid epithelial cells Hthy-ori3-1 and thyroid cancer cell lines 8305C, BCPAP, 8505C, FTC133, K1, and TPC-1 and were kindly provided by Dr. Haixia Guan (The First Affiliated Hospital of China Medical University, Shenyang, China). C643 was gifted from Dr. Lei Ye (Ruijin Hospital, Shanghai, China). Cells were routinely cultured at 37 °C in RPMI 1640 medium with 10% fetal bovine serum. KA (Cat#:57710-57-3, Cayman Chemical, UAS) was dissolved in dimethyl sulfoxide (DMSO), and stored at −20 °C until further use. Cells were treated with KA at the indicated concentrations and time points, while the same volume of DMSO was used as the vehicle control.

### Cell proliferation assay

Cells (600–1000/well) were seeded into 96-well plates for 24 h incubating, then cells were treated with different concentrations of KA for 96 h. The MTT (Sigma, Saint Louis, MO) assay was then carried out to assess the effect of KA on cell viability, and IC50 values were calculated. The plates were then read on a microplate reader using a test wavelength of 570 nm and a reference wavelength of 670 nm. Triplicates were performed for each data point.

### Colony formation assay

Colony formation assay was performed using monolayer culture. Cells (500–1000/well) were seeded into 12-well plates and treated with the indicated concentrations of KA and the medium was refreshed every 2 days. After 12 days of culture, surviving colonies (≥50 cells per colony) were fixed with methanol and stained with 0.5% crystal violet, and the colonies were then counted. Each experiment was performed in triplicate.

### Apoptosis assay

After treated with the indicated concentrations of KA for 48 h, 8505C, TPC-1, K1, C643 cells were harvested, washed with PBS, and subjected to sequential staining with Annexin V-FITC/PI Detection Kit (Roche Applied Science, Penzberg, Germany) by flow cytometer according to the manufacturer’s protocol. Early apoptotic cells show Annexin V-FITC+/PI− staining patterns, whereas late apoptotic cells exhibit Annexin V-FITC+/PI+ staining patterns. They were collectively called apoptotic cells. Each experiment was performed in triplicate.

### Seahorse XF glycolysis stress test

20000 cells were incubated in Seahorse XF96 microplates with 4 µM KA or same volume of DMSO as control for 24 h and the Seahorse XFe/XF Analyzer were turned on for warming up to stabilize. Hydrate a sensor cartridge in Seahorse XF Calibrant at 37 °C in a non-CO_2_ incubator overnight. Cell washed twice with base medium (Seahorse bioscience) containing 2 mM glutamine, and incubated for 1 h without CO2, following three baseline ECAR (extracellular acidification rate) measurements, then 10 mM glucose (activating glycolysis), 0.5 mM oligomycin (suppressing mitochondrial ATP production and shifts the energy production to glycolysis), 50 mM 2-deoxy-glucose (2-DG, inhibiting glycolysis) were injected into cells in sequence. The XF instrument directly measures the acidification rate and reports this as ECAR, which is the standard assay for measuring glycolytic function in cells.

### Glycolytic metabolites product assay

Lactic acid was measured using a commercially Lactic acid assay kit (Cat#: BC2235, Solarbio Company Beijing, China). After treated with the indicated concentrations of KA for 24 h, cells were completely cleaved by ice bath ultrasonic method, and centrifuged at 4 °C for 10 min at 12000 × *g*. The secreted Lactic acid concentration in the supernatant was determined by microplate reader at 570 nm wavelength and protein concentration were determined by Bradford method.

### Lactic acid rescue test

In colony formation assay, 4 μM KA was added to the medium separately or simultaneously with 5 mM Lactic acid (DL-Lactic acid Cat#: 50-21-5, Aladdin). The medium was refreshed every 3 days. After 12 days of culture, surviving colonies were fixed with methanol and stained with 0.5% crystal violet, and the colonies were then counted.

### ATP determination

For ATP measurement, a commercially available firefly luciferase assay kit (Beyotime Institute of Biotechnology, China) was used. Briefly, cells were incubated with indicated concentrations of KA for 24 h. After a single wash with ice-cold PBS, cells were lysed with the ATP-releasing reagent provided by the kit. Then, Luciferin substrate and luciferase enzyme were added and bioluminescence was assessed by a fluorescence spectrophotometer. The level of cellular ATP was converted to percentage of control.

### Western blot analysis

The indicated cells (4–5 × 10^5^/well) were seeded in 6-well plates and cultured for 24 h, then cells treated with or without 4 μM KA. Subsequently, cells were lysed in RIPA buffer containing protease inhibitors. Supernatants were collected and subjected to 10% SDS-PAGE, and transferred onto PVDF membranes. The membranes were then incubated overnight with primary antibodies. The following antibodies were used: Anti-E-cadherin (Cat#:76055, Abcam Biotechnology), Anti-Vimentin (Cat#:8978, Abcam Biotechnology), Anti-t-ERK (Cat#:4695, Cell Signaling Technology), Anti-p-ERK (Cat#:4370, Cell Signaling Technology), Anti-Caspase-3 (Cat#:9662, Cell Signaling Technology), Anti-Bcl-2(Cat#: WL01556, Wanleibio, China), Anti-Bax (Cat#: 50599-2-Ig, Proteintech), Anti-β-Actin (Cat#: BS6007M, Bioworld Technology), and Anti-Ki-67(Cat#:550609, BD Pharmingen).

### Caspase 3 activity assay

Caspase 3 activities were measured using a commercially Caspase 3 Activity assay kit (Cat#: C1115, Beyotime Biotechnology Shanghai, China). After treated with the indicated concentrations of KA for 24 h, cells were fully lysed and added the Ac-DEVD-*p*NA (2 mM) to test sample. After Incubated at 37 °C for 120 min, the Caspase 3 activities of cells were measured by microplate reader at 405 nm wavelength and protein concentration were determined by Bradford method.

### Xenograft tumor assay in nude mice

Female athymic nude mice were purchased from SLAC laboratory Animal Co., Ltd (Shanghai, PR. China) and housed in a specific pathogen-free environment. C643 cells (3 × 10^6^) were injected subcutaneously into the flanks of mice at the age of 5 weeks. When tumors grew to 5 mm in diameter, mice were randomly divided into control group and KA treatment group, which administered same volume of DMSO or 1 mg/kg of KA respectively through intraperitoneal injection daily for 7 days. Tumor sizes ere measured with calipers every 2 days and calculated by the formula: (length × width^2^ × 0.5). All mice were sacrificed and tumors were collected and weighted after KA treatment for 7 days. Tumor tissues were embedded in paraffin, sectioned at 4 μM, and stained with hematoxylin and eosin (H&E). Cell proliferation ability was assessed by quantification of Ki-67 staining (percentage of positive cells). In addition, H&E staining of liver and kidney sections were performed to evaluate the adverse drug reactions.

### Statistical analysis

The proliferation, colony forming ability, apoptosis, ECAR, lactic acid inhibition rate, ATP deprivation in thyroid cancer cell and the growth of xenograft tumor in nude mice between different groups were analyze by two-tailed Student’s *t* tests and chi-square test using GraphPad Prism 5 (GraphPad Software). All experiments were carried out with at least three biological replicates, while statistical significance was set at *p* < 0.05.

## Results

### KA inhibits malignant behavior of thyroid carcinoma

**Fig. 1:**
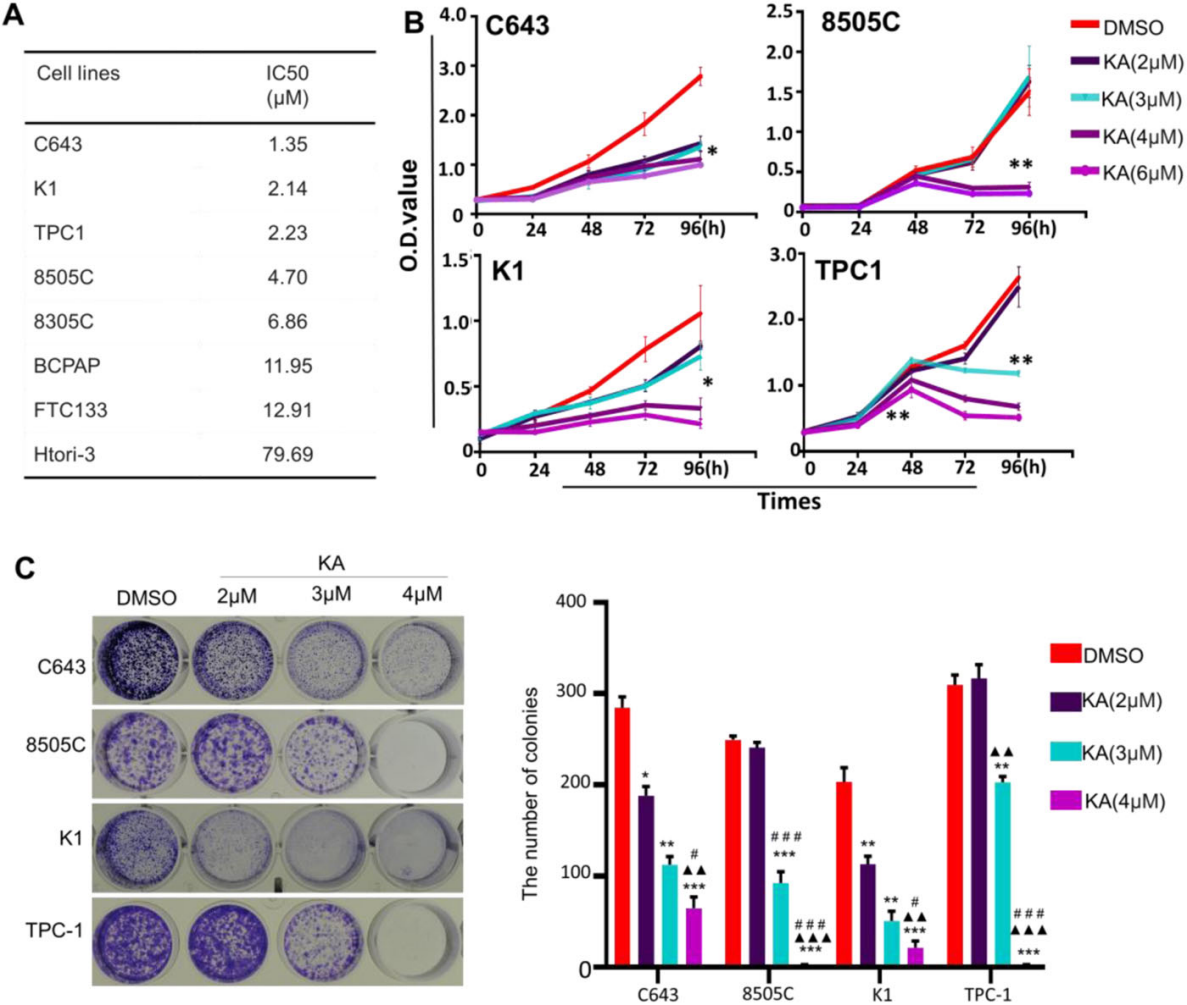
KA inhibits cell growth and colony formation capacity. **A** Thyroid cancer cell lines were treated with the indicated concentrations of KA for 24 h, followed by MTT assay to evaluate cell growth. IC50 values were calculated using the Reed–Muench method. **B** Selected four thyroid cancer cell lines were treated with the indicated concentrations of KA or vehicle control (DMSO) at the indicated time point, followed by MTT assay to evaluate the time course of cell proliferation. **C** Representative images of colony formation in 8505C, TPC-1, K1, and C643 cells treated with vehicle control (DMSO) or KA at the indicated concentrations are shown individually. Quantitative analysis of colony numbers is shown in right panel. Data are presented as mean ± SD of values from three different measurements. Statistically significant differences are indicated: *p < 0.05; **p < 0.01; ***p < 0.001 for comparison with control; ^▲▲^*p* < 0.01; ^▲▲▲^*p* < 0.001 for comparison with KA in 2 μM concentration; ^#^*p* < 0.05; ^##^*p* < 0.01; ^###^*p* < 0.001 for comparison with KA in 3 μM concentration

**Fig. 2:**
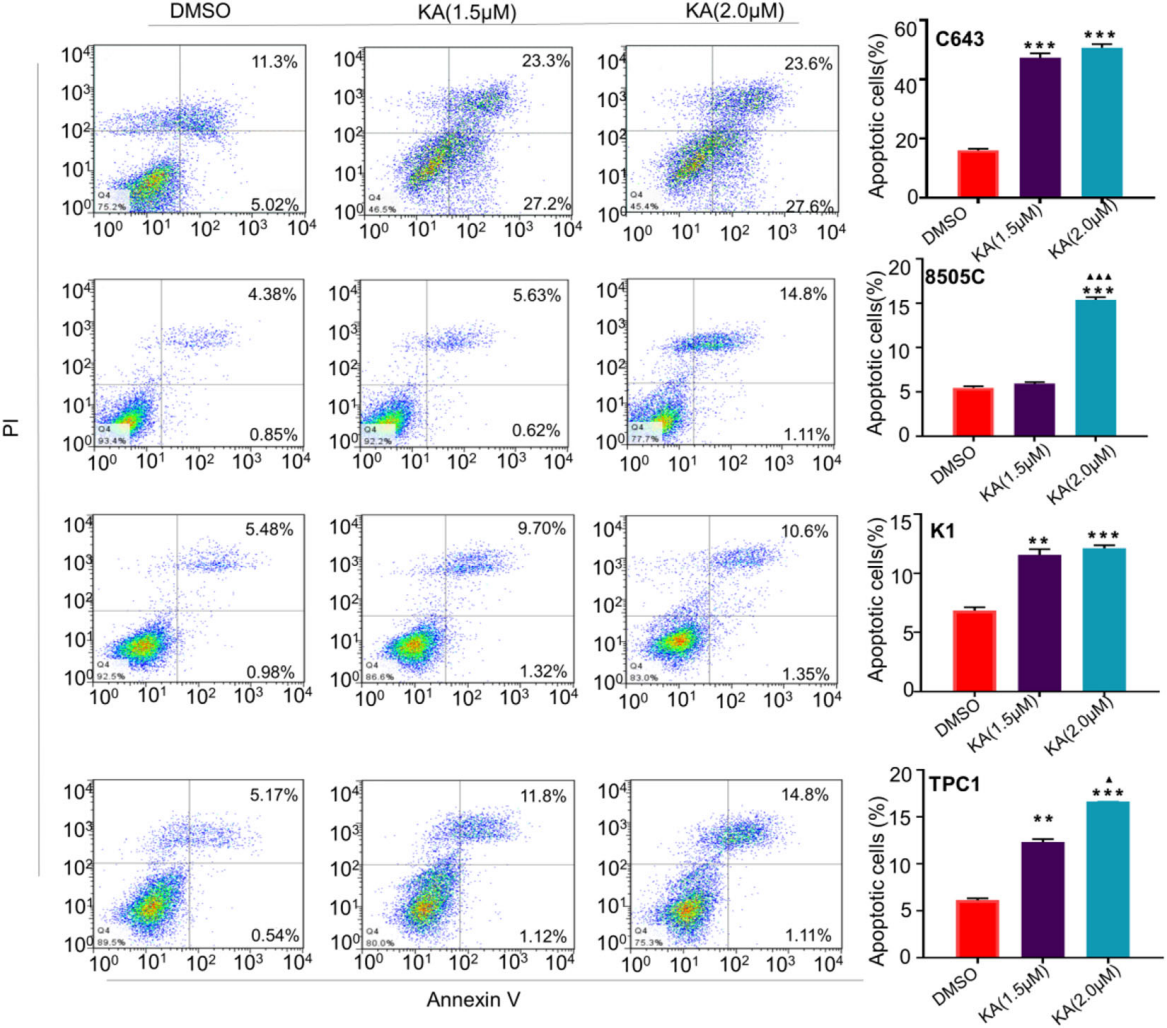
Induction of apoptosis by KA in thyroid cancer cell lines. C643, 8505C, K1, and TPC-1 cells were treated with vehicle control (DMSO) or KA at the indicated concentrations for 48 h. The percentage of early apoptotic (bottom right quarter) and late apoptotic (top right) cells is resented in the figures (left panel). After treated with KA for 48 h, 2 μM KA induced a dramatic increase in both early and late apoptosis in four thyroid cancer cell lines compared to the control. The data are presented as mean ± SD of values from three independent experiments in the right panel. Statistically significant differences are indicated: *p < 0.05; **p < 0.01; ***p < 0.001 for comparison with control. ^▲▲^*p* < 0.01; ^▲▲▲^*p* < 0.001 for comparison with KA in 1.5 mM concentration

To elucidate the antineoplastic activity of KA, we performed the MTT assay to determine the effect of KA on cell proliferation in 7 thyroid cancer cell lines and an immortalized thyroid epithelial cell HTori-3. Our results showed that KA significantly inhibited cell proliferation in a dose-dependent manner, while IC_50_ values ranging from 1.35 to 79.69 μM, especially in C643 cell line, which was more sensitive to KA treatment than other cell lines (Fig. 1A). Next, we analyzed the time-dependent response of KA in a panel of thyroid cell lines, including C643,8505C, K1and TPC-1 (Fig. 1B). Our data showed that KA can effectively inhibit the growth of thyroid cancer cell after treated for 96 h. In the meantime, we tested the effect of different concentrations of KA on colony formation capacity in four thyroid cancer cell lines (Fig. 1C). KA significantly inhibited colony forming ability in monolayer culture compared to the control, and the inhibiting ability became more significant with the increased KA concentration. As shown in Fig. 2, after treated with KA for 48 h, 2 μM KA induced a dramatic increase in both early and late apoptosis in four thyroid cancer cell lines compared to the control.

### KA weaken the glycolytic ability of thyroid cancer cells

**Fig. 3:**
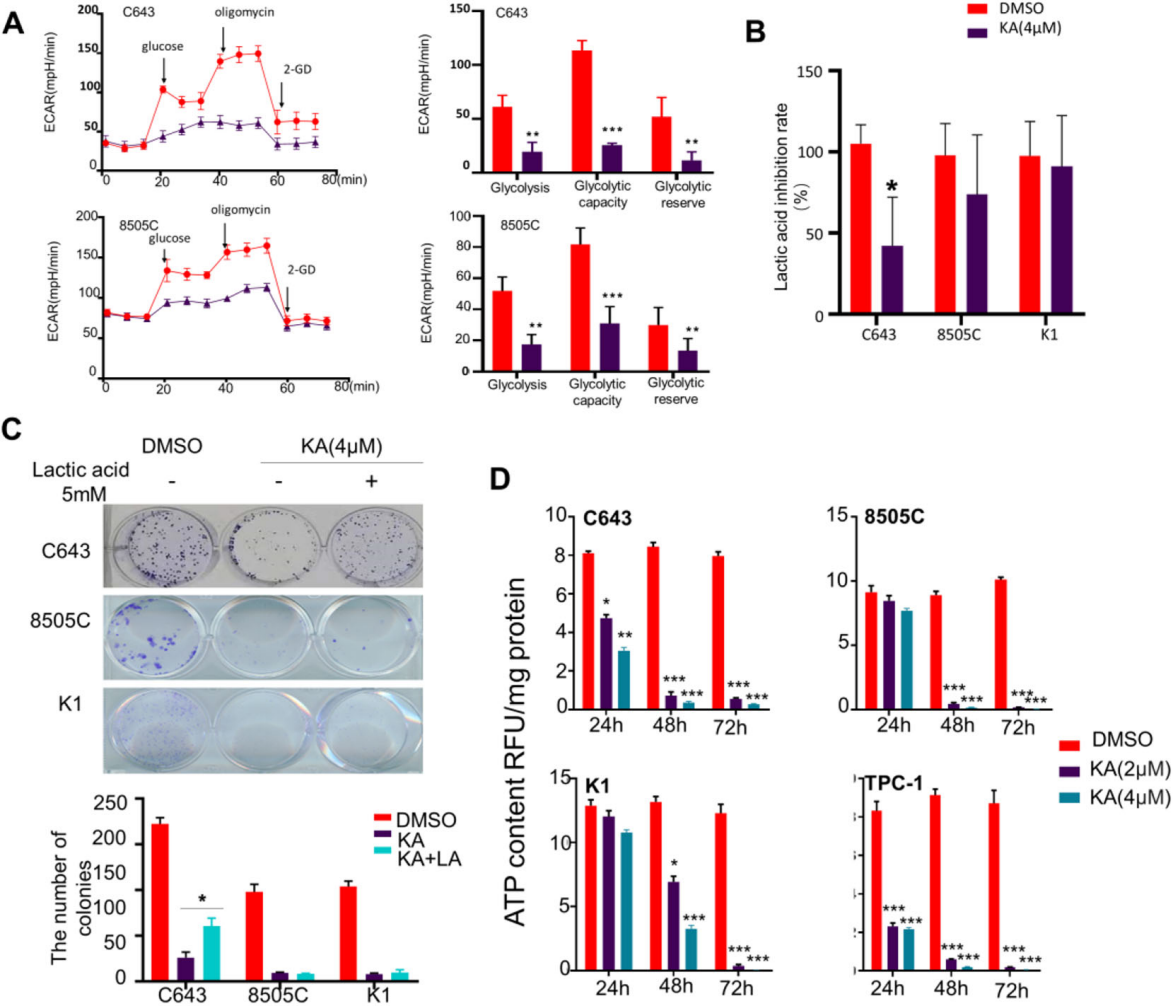
KA weaken the glycolytic ability of thyroid cancer cells. **A** ECAR is a standard and comprehensive method to assess the key parameters of glycolytic capacity. The Glycolytic Capacity of C643 and 8505C were significantly decrease after treated with KA compared with the control group. **B** After KA treatment for 24 h, the lactic acid production of thyroid cancer cell line C643 was significantly reduced, while 8505C and K1 cell lines were not. **C** 5 mM Lactic acid was added to the medium separately or simultaneously with 4 μM KA. 5 mM L-lactic acid could partially alleviate the antiproliferative activity of KA just only in C643 cell line. **D** The ATP levels experienced a rapid, extensive decrease in four thyroid cancer cell lines after treated with KA, while with the higher KA concentrations, accompanied by the more ATP deprivation. Data are presented as mean ± SD of values from three different measurands. Statistically significant differences are indicated: *p < 0.05; **p < 0.01; ***p < 0.001 for comparison with control

The Seahorse XF Glycolysis Stress Test is the standard assay for measuring glycolytic capacity in vitro. By directly measuring the extracellular acidification rate (ECAR; Fig. 3A), it provides a standard and comprehensive method to assess the key parameters of glycolytic flux: Glycolysis, Glycolytic Capacity and Glycolytic Reserve, especially in Glycolytic Capacity. After added with Oligomycin, which inhibits mitochondrial ATP production and shifts the energy production to glycolysis, the Glycolytic Capacity of C643 and 8505C were significantly decreased after treated with KA compared with the control group.

Glucose is known to be converted into pyruvate in cells, and the increased transition of pyruvate to lactic acid under anoxic conditions is called glycolysis. As mentioned above, the determination of ECAR revealed that the glycolysis ability of thyroid cancer cells was significantly inhibited by KA, and then we detected the lactic acid production of thyroid cancer cells. After KA treatment for 24 h, the lactic acid production in C643 was significantly reduced, while 8505C and K1 cells were not (Fig. 3B).

We have proved that KA treatment can significantly reduce the glycolytic ability of thyroid cancer and the lactic acid production of C643 cell was significantly reduced after KA treatment. As an end product of glycolysis, we speculated the supplementation of lactic acid could alleviate the antiproliferative activity of KA. For clone formation test, 5 mM lactic acid could partially alleviate the antiproliferative activity of KA in C643 cell, but the clone formation ability in 8505C and K1 cells failed to be rescued by adding lactic acid (Fig. 3C).

The energy demand of tumor cells depends more on glycolysis rather than aerobic oxidation. We have been previously verified that KA can significantly inhibit the glycolysis ability of thyroid cells, and then we measured the ATP level of thyroid cancer cell lines with KA treatment. As shown in Fig. 3D, the ATP levels experienced a rapid, extensive decrease in four thyroid cancer cell lines after treated with KA for 24 h, while with the higher KA concentrations, the more ATP deprivation level was observed.

### KA inhibits EMT and MAPK/ERK pathway in thyroid cancer cells

**Fig. 4:**
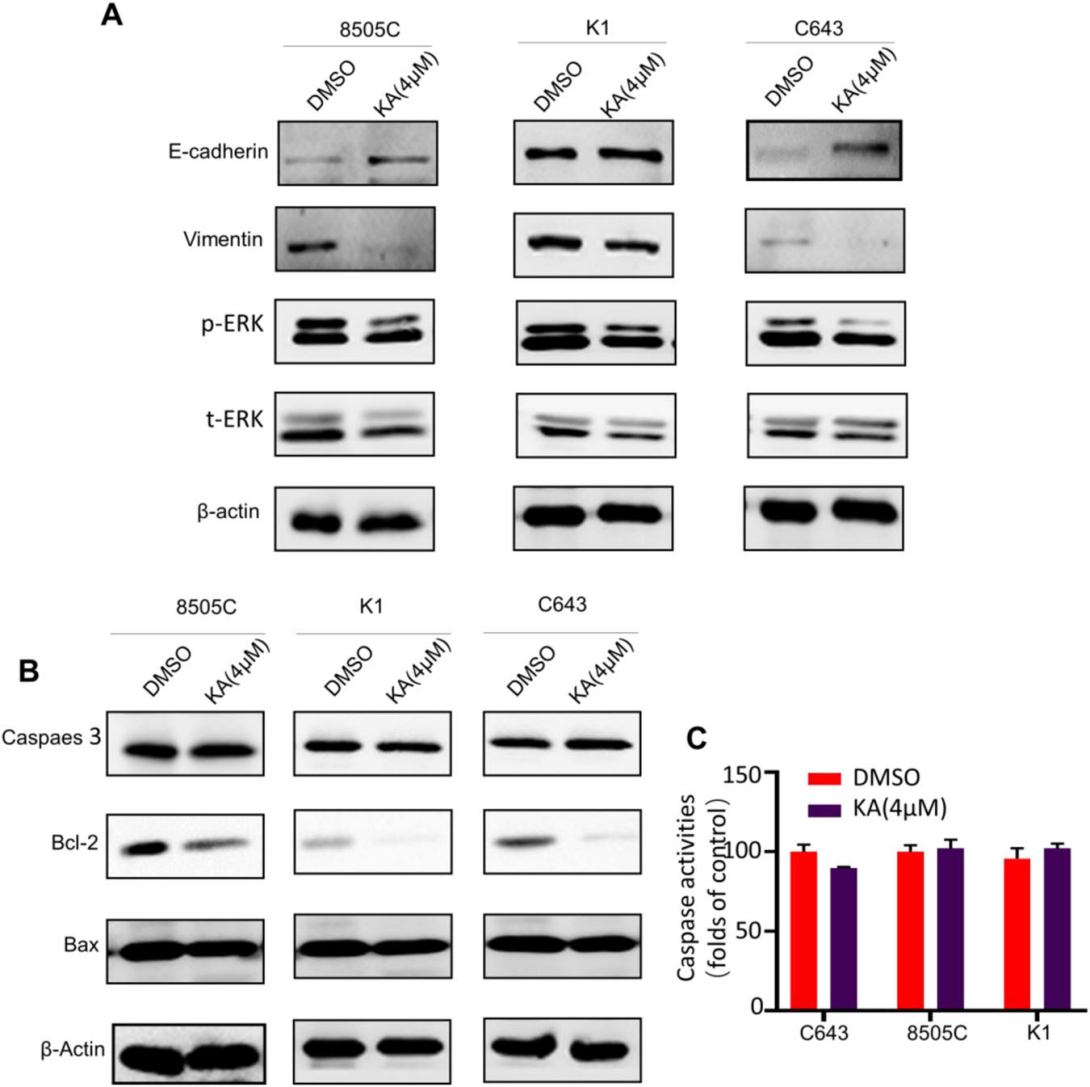
KA inhibits EMT, MAPK/ERK pathway and BCL-2 in thyroid cancer cells. **A** E‐cadherin was substantially upregulated, however Vimentin were significantly downregulated, with the following of reduced phosphorylation of ERK level in the KA-treated group compared with control. **B** The promoting apoptotic molecules Caspase 3 and Bax expression did not changed significantly, but the expression of Bcl-2 experienced downregulated significantly after treated with KA for 24 h. **C** There was no significant change in the activity of Caspase 3 after KA treatment in different cancer cee lines

Epithelial–mesenchymal transition (EMT), which plays a critical role in tumor invasion and metastasis. Given that tumor metastasis is a leading cause of death in thyroid cancers, we next tested the expression of the EMT marker E‐cadherin and Vimentin in C643, 8505C, and K1 by western blot assays. As shown in Fig. 4A, E‐cadherin was substantially upregulated, however Vimentin were significantly downregulated, following a reduced phosphorylation level of ERK in the KA-treated cells compared with control.

### KA induced apoptosis by inhibiting Bcl-2

Data from previous studies suggested that KA can significantly induce apoptosis in thyroid cancer cell lines, so we detected the apoptosis-related proteins. The results showed that the expression of promoting apoptotic molecules Caspase 3 and Bax did not change significantly, but the expression of Bcl-2 experienced significant down regulation (Fig. 4B). At the same time, we detected the activity of Caspase 3 by ELISA, and the results also confirmed that there was no significant change in the activity of Caspase 3 in thyroid cancer cell lines after KA treatment (Fig. 4C).

### KA inhibits xenograft tumor growth

**Fig. 5:**
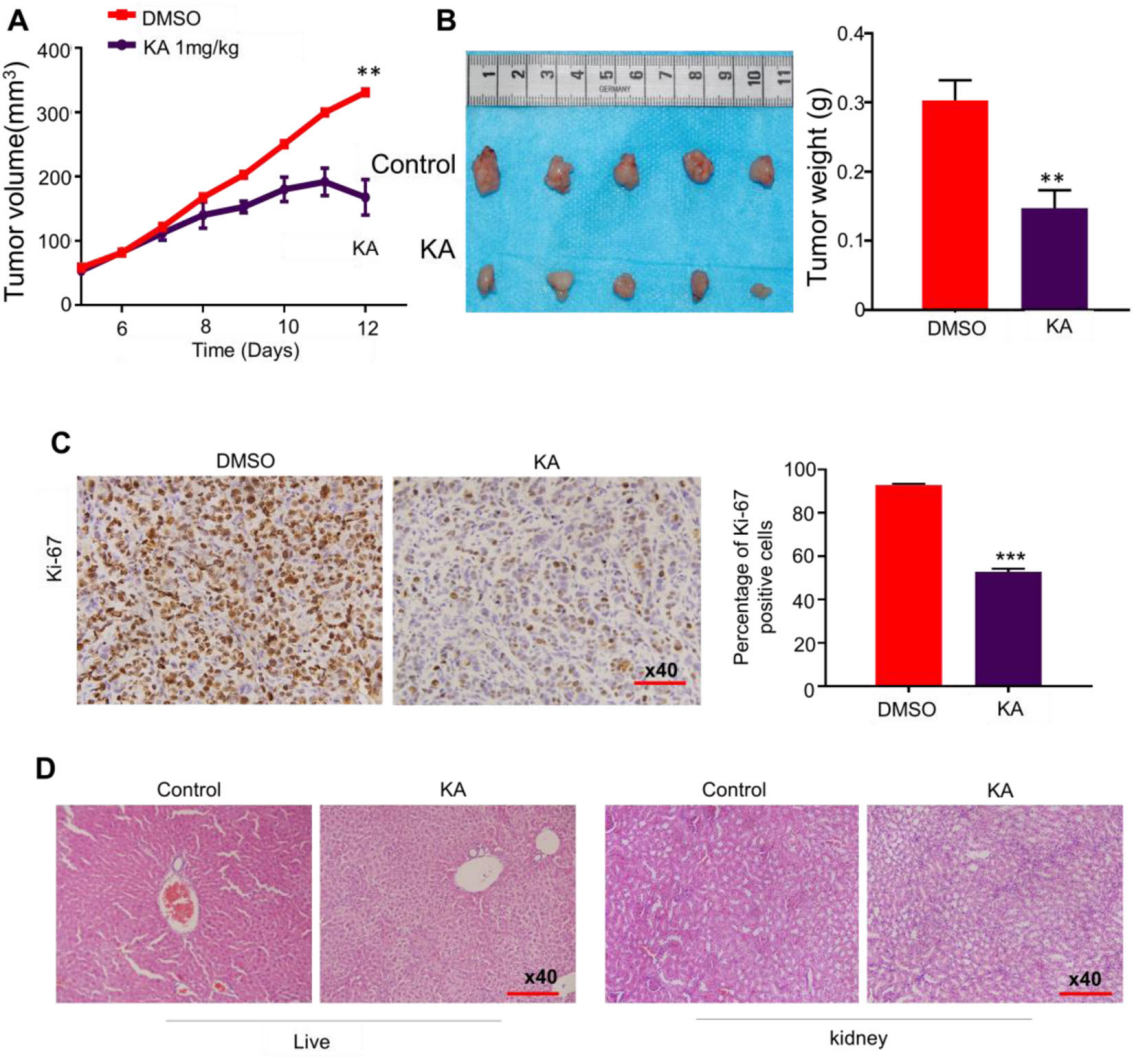
Inhibition the growth of C643-derived xenograft tumor by KA. **A** Time course of tumor growth was measured in each group at the indicated time points of various treatments. **B** Pictures were tumor weight at the end time points of different treatments. Bar graphs represents mean tumor weight from mice with the indicated treatments. **C** Representative Ki-67 stain sections of xenograft tumors. Bar graphs represent mean ± SD of the numbers of Ki-67-positive cells from five microscopic fields in each group (right panel). **D** Representative H&E stain liver and kidney sections from the indicated mice. Statistically significant differences are indicated: *p < 0.05; **p < 0.01; ***p < 0.001 for comparison with control

Given the potent inhibitory effects of KA on thyroid cancer cell growth in vitro, it is reasonable to assume that KA would be effective in treating thyroid cancers in vivo. We tested the effect of KA on the growth of xenograft thyroid tumors in nude mice. As shown in Fig. 5A, B, C643 cell-derived xenograft tumors grew progressively in the control group, whereas the tumors were slow-growing in the KA-treated group. At the end of experiments, tumors were isolated and weighted. Tumor volume and weight were significantly lower in KA-treated group compared with the control group (*P* < 0.01). We evaluated the effect of KA on cell proliferation in tumor tissues from xenograft tumor models by testing the cell proliferation marker Ki-67. Our data showed that the percentage of Ki-67 positive cells was remarkably decreased in the KA-treated group compared to the control group (Fig. 5C). Histopathological evidence demonstrated that both control (DMSO) and KA-treated hepatic tissues showed large amount of normal polygonal cells with prominent round nuclei and eosinophilic cytoplasm as well as a few spaced hepatic sinusoids arranged between the hepatic cords. No obvious proliferation of mesangial cells or severe inflammatory cell infiltration was observed in renal tissues for both groups by HE staining (Fig. 5D). Unfortunately, one mouse’s kidney showed mild mesangial cell necrosis or glomerular atrophy. There was no significant weight loss and vital organs damage in all mice after treatment. In summary, our results further supported the antitumor activity of KA in thyroid cancer, at least in xenograft tumor models.

## Discussion

Current clinical treatment of surgical thyroidectomy with adjuvant ablation by radioiodine treatment is still not satisfactory for ATC and thyroid tumors with metastasis and recurrence [3–5]. Until now, the use of multimodality strategies has failed to improve clinical outcomes significantly in those patients. Therefore, it is urgent to find an effective treatment for them.

Warburg effect refers to the fact that most tumor cells rely on aerobic glycolysis instead of aerobic oxidation, although its glycolysis yields less amount of ATP (18 times lower) compared to mitochondrial oxidation, but gives many advantages to rapidly growing tumor cells [15], so targeting metabolic pathways may be a promising approach for oncotherapy. Previous studies have confirmed that the expression levels of glycolysis-related proteins differ between thyroid cancer subtypes and are correlated with poorer prognosis, depending on the subtype [16]. The combination of glucose restriction with the antioxidant N-acetylcysteine significantly reduced ATC cell growth in vivo and in vitro. We speculate that thyroid tumor may be a glycolysis-dependent tumor, which may be one of the theoretical basis of KA treatment of thyroid cancer.

KA, to be a selective inhibitor of glyceraldehyde 3-phosphate dehydrogenase (GAPDH), a key enzyme in the glycolysis pathway, can selectively kill hyper-glycolytic cells through glucose-dependent ATP deprivation [17]. It has been reported that KA is only effective in high-glycolytic cells otherwise alternate starvation-induced metabolic pathways may be initiated to prolong tumor cell life [18]. Studies have confirmed the antitumor proliferation activity of KA in many cancer cell lines, such as A549 (lung cancer), HCT116 (colorectal cancer), KG1 (leukemia) and A375 (melanoma) [11], while the antineoplastic activity of KA in thyroid cancer in not clear.

Firstly, we confirmed that KA can significantly inhibit proliferation and clone formation ability, meanwhile induce apoptosis of thyroid cancer cell, with a dose-dependent effect. Subsequently, we used the ECAR and Lactic acid concentration assay kit to measure the of glycolytic metabolism capacity in thyroid cancer cells after treatment. The research findings show that the glycolysis ability of C643 and 8505C was significantly reduced after receiving KA. It was speculated that KA could impede the malignant behavior of tumor cells by inhibiting the glycolysis ability of tumor cells. However, the lactic acid production and clone-forming ability were significantly inhibited and rescued by supplemented with lactic acid just only in C643 cell line, but not in 8505C and K1 cell lines. It is speculated that the C643 cancer cells have higher glycolytic capacity than other cell lines. Along with inhibition of glycolysis, the thyroid cancer cell lines experienced a rapid, extensive ATP deprivation, while reduced energy supply leading to rapid increasing apoptosis after KA treatment.

As known to all, the prognosis of thyroid cancer is good generally, but not in ATC and carcinoma with distant metastasis. Given that tumor metastasis is a leading cause of death in thyroid cancer, we tested the process of EMT, which activation play critical roles in tumor invasion and metastasis. KA could significantly up-regulate the expression of E-cadherin and down-regulate the expression of Vimentin, so we speculated that KA could effectively inhibit the invasion and metastasis of thyroid cancer.

Normally, activation of a tyrosine kinase leads to a cascade of sequential phosphorylation of RAS, BRAF, MEK and then MAPK, so the MAPK/ERK pathways inhibitors have become an important target for thyroid tumors treatment [19, 20]. Given the central role of MAPK/ERK signaling pathway in thyroid tumorigenesis and malignant progression, we investigated the effect of KA on this pathway to elucidate its antitumor mechanisms. The results demonstrated that KA markedly blocked the MAPK/ERK pathways. Other studies have also shown that KA selectively kills high-glycolytic cells through glucose-dependent active ATP deprivation, and cell growth inhibition is associated with severe depletion of ATP [18]. We also confirmed that KA induced apoptosis of thyroid cancer cells is mainly related to Bcl-2 signaling pathway, but not related to caspase-3. At last, the experiments in vivo confirmed that KA inhibited the xenograft tumor growth without significant liver and kidney injury.

In summary, both in vivo and in vitro experiments confirmed that KA has effective antitumor capacity agent against thyroid cancer without severe adverse drug reactions. We have preliminarily elucidated the molecular mechanism of its potential antitumor activity. These encouraging preliminary results suggested that KA may be a promising therapeutic agent for thyroid cancer.
